# Cross-Cutting Lessons Learned During the COVID-19 Pandemic—the Walter Reed Army Institute of Research Experience

**DOI:** 10.1093/milmed/usab438

**Published:** 2021-12-04

**Authors:** Jeffrey M Osgood, Jeffrey W Froude, Sherri P Daye, Oscar A Cabrera, Matthew R Scherer, Vincent F Capaldi, Nelson L Michael, James E Moon, Eric D Lombardini, Sheila A Peel, Karen P Peterson, Deydre S Teyhen, Clinton K Murray, Robert J O’Connell

**Affiliations:** Walter Reed Army Institute of Research, Silver Spring, MD 20910-7500, USA; Walter Reed Army Institute of Research, Silver Spring, MD 20910-7500, USA; Walter Reed Army Institute of Research, Silver Spring, MD 20910-7500, USA; US Army Medical Research Directorate-West, Walter Reed Army Institute of Research, Joint Base Lewis-McChord, WA 98433-9500, USA; US Army Medical Research Directorate-Georgia, Walter Reed Army Institute of Research, Tbilisi, Georgia; Walter Reed Army Institute of Research, Silver Spring, MD 20910-7500, USA; Walter Reed Army Institute of Research, Silver Spring, MD 20910-7500, USA; Walter Reed Army Institute of Research, Silver Spring, MD 20910-7500, USA; Armed Forces Research Institute of Medical Sciences, Walter Reed Army Institute of Research, Bangkok 10400, Thailand; Walter Reed Army Institute of Research, Silver Spring, MD 20910-7500, USA; Walter Reed Army Institute of Research, Silver Spring, MD 20910-7500, USA; Office of the Surgeon General, Headquarters Department of the Army, Falls Church, VA 22041-3258, USA; Walter Reed Army Institute of Research, Silver Spring, MD 20910-7500, USA; Walter Reed Army Institute of Research, Silver Spring, MD 20910-7500, USA

## Abstract

**Introduction:**

At the start of the coronavirus disease 2019 (COVID-19) pandemic, Walter Reed Army Institute of Research (WRAIR) mobilized to rapidly conduct medical research to detect, prevent, and treat the disease in order to minimize the impact of the pandemic on the health and readiness of U.S. Forces. WRAIR’s major efforts included the development of the Department of Defense (DoD) COVID-19 vaccine candidate, researching novel drug therapies and monoclonal antibodies, refining and scaling-up diagnostic capabilities, evaluating the impact of viral diversity, assessing the behavioral health of Soldiers, supporting U.S. DoD operational forces overseas, and providing myriad assistance to allied nations. WRAIR personnel have also filled key roles within the whole of government response to the pandemic. WRAIR had to overcome major pandemic-related operational challenges in order to quickly execute a multimillion-dollar portfolio of COVID-19 research. Consequently, the organization learned lessons that could benefit other leaders of medical research organizations preparing for the next pandemic.

**Materials and Methods:**

We identified lessons learned using a qualitative thematic analysis of 76 observation/recommendation pairs from across the organization. These lessons learned were organized under the Army’s four pillars of readiness (*staffing, training, equipping*, and *leadership development*). To this framework, we added *organizing* and *leading* to best capture our experiences within the context of pandemic response.

**Results:**

The major lessons learned for *organizing* were: (1) the pandemic created a need to rapidly pivot to new scientific priorities; (2) necessary health and safety precautions disrupted the flow of normal science and put programs at risk of missing milestones; (3) relationships with partners and allies facilitated medical diplomacy and advancement of U.S. national military and economic goals; and (4) a successful response required interoperability within and across multiple organizations. For *equipping*: (1) existing infrastructure lacked sufficient capacity and technical capability to allow immediate countermeasure development; (2) critical supply chains were strained; and (3) critical information system function and capacity were suddenly insufficient under maximum remote work. For *staffing and training*: (1) successful telework required rapid shifts in management, engagement, and accountability methods; and (2) organizational policies and processes had to adapt quickly to support remote staffing. For *leading and leadership development* (1) engaged, hopeful, and empathetic leadership made a difference; and (2) the workforce benefitted from concerted leadership communication that created a shared understanding of shifting priorities as well as new processes and procedures.

**Conclusions:**

An effective pandemic response requires comprehensive institutional preparedness that facilitates flexibility and surge capacity. The single most important action leaders of medical research organizations can take to prepare for the next pandemic is to develop a quick-reaction force that would activate under prespecified criteria to manage reprioritization of all science and support activities to address pandemic response priorities at the velocity of relevance.

The novel coronavirus disease 2019 (COVID-19) pandemic is one of the deadliest global health emergencies of the past century.^1,2^ The U.S. Army provided personnel and field hospitals to hard-hit locations early in the pandemic and played a key role in the leadership and logistics of emergency use authorization vaccine rollout.^3^ Additionally, the U.S. Army leveraged its robust network of medical research laboratories and personnel to support the national scientific response to the pandemic through direct scientific research and personnel support for the whole of government efforts (e.g., Operation Warp Speed; OWS). Among the six major biomedical research facilities in the U.S. Army, the largest portfolio of COVID-19 work was performed at Walter Reed Army Institute of Research (WRAIR).

WRAIR is the largest biomedical research laboratory administered by the Department of Defense (DoD) and is part of the U.S. Army Medical Research and Development Command (USAMRDC). WRAIR conducts medical research to optimize health and performance through innovative infectious disease and brain health research. The institute consists of over 2,600 personnel across dozens of locations in North America, Africa, Asia, and Europe. In addition to longstanding and diverse relationships across its footprint, WRAIR possesses critical infrastructure and expertise, including a pilot bioproduction facility (PBF), animal research capabilities, clinical diagnostic and research laboratories, and robust clinical trial capabilities. Accordingly, WRAIR has played an important role in responding to past public health crises, including HIV, Ebola, and Zika.^4^

WRAIR built on decades of strategic investments and experience to respond to the COVID-19 pandemic. WRAIR’s COVID-19 research efforts included the development of the DoD COVID-19 vaccine candidate (Spike Protein Ferritin Nanoparticle adjuvanted with Army Liposomal Formulation-QS21), development of novel drug therapies and monoclonal antibodies, refining and scaling-up military diagnostic capabilities, defining infection impacts at Army basic training locations, assessing the impact of viral diversity, and documenting the effect of the pandemic on the behavioral health of U.S. Armed forces.^5^ Additionally, WRAIR’s outside the continental United States (OCONUS) laboratories in Africa, Asia, and Europe advanced U.S. global health diplomacy through the provision of multifaceted assistance to host nations.^6,7^ WRAIR personnel also filled key roles within the White House coronavirus task force, OWS, and the World Health Organization advisory group, detailing more than a dozen personnel to support these strategic teams.

The focus of this paper is to explore cross-cutting lessons learned that impacted operations across WRAIR’s activities rather than any single effort (see Table I). We organized these lessons under the Army’s four pillars of readiness (*equipping, staffing, training*, and *leadership development*). To this framework, we added *organizing* and *leading*.

**TABLE I.** Key Lessons and Recommendations for Future Pandemic Research Readiness

Lesson learned	Recommendation(s)
*Organizing*	
The pandemic created a need to rapidly pivot to new scientific priorities.	Work with stakeholders to empower organizational leaders with authority to repurpose funds in a crisis.
Necessary health and safety precautions disrupted the flow of normal science and put programs at risk of missing milestones.	Build contingency plans with stakeholders to priority rank efforts and adjust milestones in a crisis.
Relationships with partners and allies facilitated medical diplomacy and advancement of U.S. national military and economic goals.	Anticipate approvals and agreements that would allow surveillance labs to act quickly on behalf of partners.
A successful response required interoperability within and across multiple organizations.	Build partnerships during inter-years. Clearly communicate needs and timelines with partners.
*Equipping*	
Existing infrastructure lacked sufficient capacity and technical capability to allow immediate countermeasure development.	Invest in modernized infrastructure that will focus on cell line development, high throughput manufacturability assessment, and formulation/stability.
Critical supply chains were strained.	Ensure adequate stockpile of supplies. Plan how distribution of critical supplies will be prioritized during shortages.
Critical information system function and capacity were suddenly insufficient under maximum remote work.	Ensure that there always will be adequate network capacity for full teleworking. Perform annual stress test of all information systems that support telework functions.
*Staffing and t* *raining*	
Successful telework required rapid shifts in management, engagement, and accountability methods.	Create telework agreements for all staff. Cross-train/certify staff to keep positions 2–3 people deep.
Organizational policies and processes had to adapt quickly to support remote staffing.	Adjust on-site support staffing to mirror sections they support.
*Leading and leadership development*	
Engaged, hopeful, and empathetic leadership made a difference.	Model and promote best health practices. Share new information quickly.
The workforce benefitted from concerted leadership communication that created a shared understanding of shifting priorities as well as new processes and procedures.	Standardize reporting processing, requirements, and schedules as much as possible.

Cross-cutting observations and recommendations based on Walter Reed Army Institute of Research experience conducting COVID-19 and non-COVID-19 research during the COVID-19 pandemic.

## METHODS

Section leaders from across the organization provided 3–5 lessons learned on a standardized form in response to an official tasker resulting in 76 pairs of observations and recommendations. Subsequently, the authors used thematic analysis following published best practices to manually code the data and identify common themes.^8^ Thematic analysis was chosen to facilitate participatory research and produce a maximally accessible report.^8^ We iteratively refined common categories into 11 subtheme statements organized under four major themes and validated results with member-checking. Those coding data and refining themes were personnel with oversight of all WRAIR COVID-19 activities. The observations and recommendations provided alongside each lesson learned heading were created by combining and summarizing the source content.

## LESSONS LEARNED

### Organizing

#### The pandemic created a need to rapidly pivot to new scientific priorities

##### Observations.

The pandemic created an urgency to deprioritize previously approved efforts and initiate COVID-19 research. In order to respond quickly to the crisis, the review and approval of COVID-19 research had to occur outside the normal prioritization process. Although funding arrived more rapidly than usual for Army research, the overall process was not as agile as the mechanisms used by other federal agencies. For example, the National Institute of Allergy and Infectious Diseases (NIAID) rapidly funded a phase 1 vaccine trial with Moderna; the first injection into a study volunteer occurred within 2 weeks of WHO declaring COVID-19 a pandemic.^9^ The National Institutes of Health–Moderna COVID-19 vaccine was ultimately one of the first to receive emergency use authorization from the U.S. Food and Drug Administration.

To facilitate the new research mission, WRAIR streamlined operations supporting agreements, human subject’s protection review, and public health response procedures. However, as programs were initiated, there was frequent and multilayered oversight in the early phases of countermeasure development, which absorbed bandwidth that might have otherwise been directed toward research execution.

WRAIR experienced the most success in areas where the organization was able to shift resources and leverage flexibility in contracts and agreements to support COVID-19 work quickly. Efforts were more likely to result in deliverables at the velocity of relevance in cases where only minimal secondary review and authorization were required from stakeholders. In contrast, scientific efforts that required extensive oversight at multiple levels were less likely to create translatable solutions faster than they became available from other federal agencies and/or private industry.

##### Recommendations.

Leaders and stakeholders should work together to establish emergency courses of action (COAs) to empower senior organizational leaders with authority to quickly reprioritize and reallocate research and funding to respond to pandemic priorities while awaiting dedicated funds. One way to implement this would be for organizational leaders to delegate this authority to a medical research quick-reaction force (QRF) comprised of section leaders and experts from across the organization. The goal should be to pair flexibility in agreements, contracts, and funding with contingency plans and empowered leaders at the lab level to reallocate funds and commence work on emergency efforts within days during a crisis. Although this approach involves programmatic risk, there is also significant risk to programs, military readiness, and public health in delaying research during a pandemic. This approach may work best for organizations with deep institutional knowledge about responding to emergent health crises; however, organizations without this experience may require more oversight.

#### Necessary health and safety precautions disrupted the flow of normal science and put programs at risk of missing milestones

##### Observations.

National, state, and local governments imposed significant health-protective actions (e.g., lockdowns). Similarly, WRAIR implemented policies to protect staff and research volunteers (e.g., restricting on-site personnel). Although these efforts were necessary, they disrupted research operations and created issues achieving program milestones. Research teams had to choose which projects to prioritize, and numerous research efforts were either canceled or postponed. WRAIR sections worked quickly to address this risk. In some cases, sections had emergency response plans, which included a priority list, which investigators refined during the early months of COVID-19. For other affected projects, lab sections worked with stakeholders to adjust milestones.

##### Recommendations.

Foster internal and stakeholder engagement before the emergence of a pandemic to create contingency plans for disruption of regular research operations and to adjust program requirements appropriately. Plans should be updated regularly, and teams should develop and maintain priority lists of projects to serve as a starting point for emergency reprioritization of resources.

#### Relationships with partners and allies facilitated medical diplomacy and advancement of U.S. national military and economic goals

##### Observations.

WRAIR’s OCONUS labs, operating under county-specific Chief of Mission authorities, advanced diplomatic initiatives that promoted partnership building and sustainment, host nation capability development, and improved local health outcomes. The myriad support provided by WRAIR’s OCONUS directorates included subject matter expertise and training, direct surveillance, basic research, preclinical work, and regulatory support. In aggregate, WRAIR’s OCONUS support for host nations during the pandemic provided local officials and U.S. diplomats the opportunity to acknowledge U.S. support for their successful management of the pandemic.^6,7^ Years of partnerships between WRAIR’s OCONUS directorates and host nations’ governments facilitated these efforts. Indeed, WRAIR OCONUS directorates serve as infectious disease surveillance networks that can rapidly pivot to emerging infectious disease outbreaks. Accordingly, WRAIR performed extensive COVID-19 testing across its sites in Europe, Africa, and Asia. For example, WRAIR’s OCONUS sites performed over 85 K tests by the 1 year anniversary of the WHO declaration of the COVID-19 pandemic. Furthermore, OCONUS directorates received partner requests for funding to support the detailed characterization of clinical samples to identify SARS CoV-2 variants.

Although impactful, the enterprise-wide diagnostic testing response was not immediate and the authority to initiate host nation support at the WRAIR OCONUS directorates was developed through an iterative process of local national public health requests, Chief of Mission approvals, and coordination with senior approval authorities. Regional variability in pandemic severity, host country needs, and OCONUS lab capability necessitated country-specific coordination. All forward sites were eventually granted authorization to perform testing through a DoD-level exception to policy allowing limited use of surveillance testing capacity for diagnostic testing on samples from local nationals. A standing, broadly applicable exception to policy memorandum or similar mechanism responsive to the need for broad outbreak response testing would have permitted immediate engagement and host nation support.

##### Recommendations.

Anticipate the requirement for prerequisite, established regulatory approvals and agreements that would allow surveillance labs to act quickly on behalf of partners and allies when the next pandemic strikes. The availability of outbreak response funding and the authority to procure test kits will be critical to ensure sustained support. In addition, consider expanding the availability of multifunctional diagnostic platforms in surveillance networks. Communicate with surveillance partners early and often on the synchronization of efforts to ensure expectations are met. Be specific regarding capabilities and expertise when communicating with partners. Local partners and staff will become invaluable when executing research at remote sites during pandemic-related restrictions. Finally, identify resources to support the augmentation of local capabilities to perform next-generation sequencing/characterization to enhance visibility on variant threats.

#### A successful response required interoperability within and across multiple organizations

##### Observations.

Responding to the COVID-19 pandemic required coordination across many organizations. USAMRDC leadership responded to this challenge with the creation of a coordination cell and synchronization meetings with representatives from each USAMRDC component. The success of many efforts also relied on partnerships with other DoD elements, the Department of Health and Human Services (HHS), the Department of State, academia, allied nations, and the private sector. In most cases, this resulted in well-coordinated activity and successful projects; however, there were also instances where potential collaborations were canceled due to a failure in aligning timelines and effectively communicating needs across organizations.

##### Recommendations.

Prioritize building partnerships with sister organizations. Such efforts will prepare for interoperability during a pandemic and broaden capabilities for normal missions during the inter-years. Moreover, when coordinating efforts, ensure that all parties have clear and realistic understandings of each other’s needs and timelines.

### Equipping

#### Existing infrastructure lacked sufficient capacity and technical capability to allow immediate countermeasure development

##### Observations.

DoD medical countermeasure discovery and development relies on the transition to commercial partners for late-stage product development. Reliance on external process development and large-scale manufacturing creates discontinuity that leads to significant schedule and performance risk for DoD-developed products. During the production of the DoD COVID-19 vaccine at WRAIR, the combination of accelerated timelines and lack of an identified commercial partner led to one failed production run and delays in manufacturing and clinical development. Pilot manufacturing facilities such as the WRAIR PBF remove some of the risk perceived by commercial partners looking for products emerging from early research and development phases. Of note, passive antibody-based medical countermeasures offer a likely more rapidly developed vaccine alternative that could be employed to counter future infectious diseases and medical concerns of the DoD.^10^ Finally, the DoD does not have a suitable mechanism for manufacturing and fill/finish capability at the pilot and early scale.

##### Recommendations.

Invest in modernized infrastructure that will focus on cell line development, high-throughput manufacturability assessment, and formulation/stability assessment to ensure DoD medical countermeasures are derisked early in the development process. In particular, the DoD will need both process development infrastructure as well as organic suspension bioreactor capacity in order to be rapidly responsive to current and future production needs of novel classes of medical countermeasures. As an alternative, the intent of this capability may be achievable through outside partnerships if agreements provide DoD with priority. Access to this equipment and capability supports a reduction of the acquisition risk by transitioning products that have an aligned and scalable technical transfer plan to advance developers and commercial partners by aligning R&D scale equipment to production scale devices within both HHS and DoD commercially partnered advanced development and manufacturing facilities.

#### Critical supply chains were strained

##### Observations.

The COVID-19 pandemic produced severe supply chain disruptions of essential research supplies across the globe. Supply shortages and vendor disruptions also delayed maintenance of research infrastructure and medical equipment. These shortages created research delays and forced prioritization of projects. The shortage of personal protective equipment (PPE) was especially challenging for our remote OCONUS locations where it took several months to procure enough PPE to resume study activities.

To respond to this challenge, organizational sections developed minimal stock levels and reordering frequency for critical supplies. Furthermore, some sections obtained pledges from vendors that they would continue to receive prepandemic levels of certain essential supplies. Elements within the organization with stockpiles of essential supplies were able to rotate these into use and minimize discontinuity of efforts. USAMRDC also established a PPE tracker early in the pandemic and assisted with obtaining supplies. This support helped mitigate the effects of the national shortage on PPE.

##### Recommendations.

Ensure enough critical supplies are on hand for at least 1 month of essential activities. Use contract language that will guarantee continued procurement at necessary levels. Plan ahead how the distribution of critical supplies will be prioritized during severe shortages. Prepare for maintenance vendor disruption by working with equipment users to identify local backup vendors and training users in basic preventive maintenance checks and services. Consider scheduling periodic maintenance earlier in the maintenance cycle to provide greater flexibility in the event of a disruption.

#### Critical information system function and capacity were suddenly insufficient under maximum remote work

##### Observations.

In order for teleworking administrative staff to optimally support the scientists who were in the labs, WRAIR had to overcome several technological challenges. Telework platforms were unreliable in the initial weeks as there was not enough capacity on the virtual private network. Furthermore, some employees did not have a government laptop. Moreover, some OCONUS labs did not have a dedicated audio bridge or web conferencing capabilities. Ultimately, these network challenges were mitigated as new tools became available. We maximized telework with Microsoft® Teams interactions through the Commercial Virtual Remote work environment, which allowed effective communication and coordination for all non-benchwork activity at WRAIR CONUS sites; however, the majority of WRAIR’s OCONUS workforce was ineligible for Microsoft® Teams accounts and had to find other platform solutions.

##### Recommendations.

Ensure that there will always be adequate capacity for full-unit teleworking on the network and that mission-critical employees will have the required hardware. Likewise, digital platforms to facilitate teleworking should be embraced and utilized before maximum telework is needed. Verify network capacity with an annual stress test of all information systems that support telework.

### Staffing and Training

#### Successful telework required rapid shifts in management, engagement, and accountability methods

##### Observations.

Similar to the global workforce, there was a need to transition to telework to minimize the risk of transmission and protect the team. This was especially true for WRAIR, as we had to protect those essential personnel whose mission was to create medical countermeasures to defeat COVID-19. The organization adopted a proactive posture early in the pandemic by maximizing the telework of nonessential personnel. In cases where on-site staff was required, sections utilized alternating schedules.

Many sections quickly and effectively adjusted to maximum telework. However, some sections lost valuable work hours because they did not have telework agreements in place for all of their staff. Relatedly, effective monitoring of health status and COVID precautions for all personnel who were on telework status proved difficult, particularly for contract employees. Furthermore, some of the mission-critical work at WRAIR could not be done remotely. For this reason, on-site staff had to expand their responsibilities to cover for those on telework. Consequently, additional training was required due to unfamiliarity with standard operating procedures; the increased workload did lead to staff burnout.

##### Recommendations.

Organizations should have agreements in place to enable maximum telework at any given time. To succeed within staffing number restrictions, strive for personnel redundancy such that coverage for key operations is at least 2–3 people deep. Cross-train and cross-certify staff routinely to ensure no loss of proficiency over time. Scheduling should stay responsive and flexible. Furthermore, include language in contracts that would facilitate location tracing and monitoring of the health status of contractors during an emergency.

#### Organizational policies and processes had to adapt quickly to support remote staffing

##### Observations.

The pandemic created sudden administrative challenges, while maximum telework disrupted standard administrative processes. Some sections responded to this very effectively. For instance, the WRAIR Institutional Review Board and Human Subjects Protection Branch acted quickly to provide guidance to researchers on how to adjust research operations to protect the health of staff and volunteers without running afoul of other regulations. The Human Subjects Protection Branch entered the pandemic with proactive measures in place that helped hasten COVID-19 research (e.g., broad screening protocols for clinical trials). Ultimately, proactive planning and responsive processes enabled expedited ethical reviews while ensuring quality and safety. This allowed a faster start to COVID-19 science and helped mitigate the impact of the pandemic on ongoing research.

##### Recommendations.

Ensure that administrative processes will be able to operate smoothly during pandemic-related disruptions and adapt to pandemic-specific needs and limitations. For instance, consider virtual in-/out-processing procedures. During maximum telework, consider a grace period for individuals with expired credentials to access buildings. On-site presence of support services staff should adjust to match the existing workload of the sections they support.

### Leading and Leadership Development

#### Engaged, hopeful, and empathetic leadership made a difference

##### Observation.

It was critical to personnel that leaders provided resources, guidance, and moral support. WRAIR leaders distributed illustrative media with guidance for staying healthy and resilient, as well as quarantine and isolation requirements.^11^ The WRAIR Commander provided daily updates to section leaders with the latest information on the pandemic and encouragement. WRAIR initiated virtual town halls with the Commander. Some leaders within the organization started weekly teleconferences to share resources, information, and ideas and to provide moral support to colleagues at WRAIR OCONUS laboratories. Similarly, many sections utilized virtual events to supplement individual counseling, conduct training, and help acclimate new personnel to the organization.

To help employees cope with the mental stress of the pandemic, WRAIR hosted several virtual events for community members to express shared challenges and hear recommendations from experts in the fields of behavioral health and infectious disease. WRAIR also implemented quarterly meetings focused on enhancing resilience. Finally, leaders highlighted positive news on testing, drug discovery, and vaccine development, which provided employees with a counterweight to the predominantly pessimistic news coverage.^12^

Data suggest that such leader actions are impactful. Indeed, a WRAIR/Army Public Health Center survey of U.S. Soldiers in North America, Europe, and Asia found that Soldiers were more likely to engage in protective health practices if their leaders engaged in COVID-19 leadership behaviors.^13,14^

##### Recommendations.

As a leader, model and promote best health practices. Provide engaged, hopeful, and empathetic leadership to your workforce. Morale is a critical component to the organizational response in an emergency, so sharing positive news is a key leader action that will pay dividends. Leverage technology to keep personnel connected and updated with the latest guidance and information about the pandemic. Group-based virtual events can assist with rapport building and cover the common ground for employee development. Frequent touch points with team members will help reduce the impact of social isolation within the workforce and reduce anxiety about expectations and productivity in a telework environment. Maintain frequent communication with field site leaders.

#### The workforce benefitted from concerted leadership communication that created a shared understanding of shifting priorities as well as new processes and procedures

##### Observations.

As the organization’s focus changed to COVID-19, the administrative teams supporting WRAIR’s COVID-19 efforts had to adopt a new shared understanding regarding roles, responsibilities, information flow, reporting requirements, and purpose of different reporting products. This change in processes and procedures were compounded as WRAIR received changing reporting requirements from multiple external stakeholders. Similar requests for information (RFIs) were sometimes unknowingly worked by several administrative teams simultaneously. At times, this increased the burden on science staff and left vital personnel out of the information loop. Initially, changes in reporting requirements consumed valuable time and led to confusion. WRAIR headquarters addressed this by standardizing information flow with new universal reporting templates, reporting chains, and battle rhythm. WRAIR also established designated individuals at each organizational level to manage reporting requirements. These efforts helped alleviate the bureaucratic burdens mentioned in the first lesson of this paper.

##### Recommendations.

Establish an administrative cell with visibility on all external-facing reporting for pandemic research. Communicate to external stakeholders that RFIs should staff through this cell. Designate reporting points of contact for all RFIs for each section to minimize distractions for science staff. Consider using shared continuity documents to clearly define roles, responsibilities, and processes. For internal reporting, establish standard reporting formats and schedules that can satisfy as many requirements as possible with minimal paperwork by science staff.

## DISCUSSION

WRAIR executed critical research on COVID-19 prevention, diagnostics, treatments, and the behavioral health impact of the pandemic. Scientists and subject matter experts impacted decision-making at the highest levels of the Army and the Federal Government. Furthermore, WRAIR leveraged its OCONUS network of field lab sites to support a robust COVID-19 response for U.S. allies and partners, which potentially strengthened geopolitical relationships. The lessons of these experiences provide opportunities to further improve the response to the next pandemic.

The above lessons share some similarities with those reported from operational units working to protect the health of their personnel.^15^ In both cases, creating a shared understanding of the unit’s response to the pandemic and providing empathetic leadership were emphasized. However, many of WRAIR’s experiences in the research arena are unique. In particular, the pandemic created a forcing function for WRAIR to rapidly pivot many of its resources away from its existing missions to execute a broad portfolio of COVID-19 work. One limitation of our paper is that lessons from nonscience sections of our organization (e.g., safety and operations) were not as well represented. Thus, future inquiries into this topic should consider those perspectives more.

WRAIR’s response to the pandemic ultimately required an institutional shift of priorities and efforts. Readiness and the ability to respond rapidly to changing circumstances and priorities predicted success. Based on this experience, the single most important action for medical research and development organizations to prepare for the next pandemic is to develop a QRF. The QRF would activate under prespecified criteria and direct reprioritization of all science and support activities to conform to pandemic priorities. The goal of the QRF should be to effect reprioritization and reallocation of funds, staff, and other resources to authorize the commencement of pandemic research activities within days of activation. The QRF should consist of senior personnel from across the organization, including research and support sections. Organizational leadership should provide clear intent as to the purpose and scope of authority of the QRF. It will be important that the QRF is empowered to act decisively by bypassing many ordinary bureaucratic processes that do not impact safety or scientific efficacy. Therefore, during the inter-years, the QRF should work with leadership, stakeholders, partners, and organizational sections to create inter-agency contingency plans.

## CONCLUSION

Military theorist John Boyd emphasized maneuverability as paramount in planning for war;^16^ this principle also applies to scientific wars on pandemics. The ability to rapidly pivot to changing priorities during a pandemic is critical to creating translatable solutions at the velocity of relevance. In other words, agreements, contracts, funding, surveillance mechanisms, and research programs must be maneuverable in a crisis. Moving forward, it will also be critical to sustain research on threats such as emerging coronaviruses and pandemic influenza after the acute crisis of SARS-CoV-2 has abated. Multiple disease outbreaks and pandemics have occurred in the past 20 years, and the risk of pandemics will persist into the future.^17^ The lessons learned during the COVID-19 pandemic may inform and assist science organizations as they prepare for the next pandemic.
